# Reappraisal—but not Suppression—Tendencies Determine Negativity Bias After Laboratory and Real-World Stress Exposure

**DOI:** 10.1007/s42761-021-00059-5

**Published:** 2021-10-22

**Authors:** Candace M. Raio, Nicholas R. Harp, Catherine C. Brown, Maital Neta

**Affiliations:** 1 Department of Psychiatry, New York University Grossman School of Medicine, New York, NY USA; 2 Department of Psychology, University of Nebraska-Lincoln, Lincoln, NE USA

**Keywords:** Stress, Reappraisal, Ambiguity, Negativity bias, COVID-19

## Abstract

Higher reactivity to stress exposure is associated with an increased tendency to appraise ambiguous stimuli as negative. However, it remains unknown whether tendencies to use emotion regulation strategies—such as cognitive reappraisal, which involves altering the meaning or relevance of affective stimuli—can shape individual differences regarding how stress affects perceptions of ambiguity. Here, we examined whether increased reappraisal use is one factor that can determine whether stress exposure induces increased negativity bias. In Study 1, healthy participants (*n* = 43) rated the valence of emotionally ambiguous (surprised) faces before and after an acute stress or control manipulation and reported reappraisal habits. Increased negativity ratings were milder for stressed individuals that reported more habitual reappraisal use. In Study 2 (*n* = 97), we extended this investigation to real-world perceived stress before and during the COVID-19 pandemic. We found that reappraisal tendency moderates the relationship between perceived stress and increased negativity bias. Collectively, these findings suggest that the propensity to reappraise determines negativity bias when evaluating ambiguity under stress.

**Supplementary Information:**

The online version contains supplementary material available at 10.1007/s42761-021-00059-5.

## Introduction

Stress exposure is pervasive in everyday life and has been widely shown to impose a number of changes in affective processing, particularly in contexts marked by uncertainty. One consequence of stress or threat exposure is that it tends to engender a “negativity bias” when evaluating ambiguity (Grupe & Nitschke, 2013; Hartley & Phelps, 2012; Lerner & Keltner, 2000; Tanovic et al., 2018). For example, higher reactivity to stress exposure (i.e., cortisol increase) is associated with more negative perceptions of ambiguous facial expressions (e.g., surprised faces; Brown et al., 2017). Similar findings have been reported in individuals with higher anxiety (Bishop et al., 2015; Park et al., 2016; Richards et al., 2002), negative affect (Ito et al., 2017), and elevated threat-induced physiological arousal (Neta, Cantelon, Mahoney et al., 2017; Neta, Cantelon, Haga et al., 2017) or amygdala activation (Neta & Whalen, 2010). These empirical reports align with a broader theoretical literature suggesting that stress and its related affective and physiological states confer a higher propensity to detect threats in ambiguous contexts (Grupe & Nitschke, 2013; Hartley & Phelps, 2012; Lerner & Keltner, 2000; Tanovic et al., 2018). These stress-related biases may be evolutionarily adaptive given that under stressful circumstances prioritization is given to ensure safety and survival. However, these biases can also lead to negative appraisals of ambiguous cues or environments and drive a range of maladaptive avoidance behaviors (LeDoux & Daw, 2018). Identifying factors that determine whether negative biases will emerge after stress exposure, or whether individuals may instead respond more adaptively to stress, may help us better understand what confers psychological resilience under stress.

Ambiguous stimuli are particularly well-suited for measuring individual differences in biases of emotional perceptions under stress since they can either signal the presence of positive or negative environmental events. Ambiguous social stimuli, such as surprised facial expressions, are canonically used in the affective science literature to test emotional biases because they can readily (and accurately) be perceived as being driven by either positive (e.g., unexpected gift) or negative (e.g., car accident) outcomes. Thus, one’s tendency to extract a negative meaning from ambiguous cues constitutes one’s “valence bias” (Neta et al., 2009). It is important to note that unlike other tasks using emotional facial expressions, valence bias tasks such as this one are intended to measure *perceptions* of emotional stimuli, rather than induce a particular emotional *experience* from viewing these emotional expressions. Although there is wide variability in valence bias across individuals, behavioral and neuroimaging studies have supported an initial, bottom-up negative appraisal of ambiguous stimuli (Neta & Tong, 2016; Petro et al., 2018). It is thought that because stress prioritizes bottom-up processing (Hermans et al., 2014; Rauch et al., 2000), it thereby contributes to a negative valence bias by shifting perceptions of emotional stimuli toward negative appraisals (Brown et al., 2017). That is, the emotional experience of stress can subsequently alter the perception of emotional ambiguity. However, the stress-negativity correspondence is not always one-to-one; some participants demonstrate increased perceptions of negativity under stress, while others demonstrate increased positivity—or no change—in how they perceive ambiguity (Brown et al., 2017). This suggests that, even in the face of the same stressor, individuals vary in how resistant they are to the effects of stress on negativity bias.

One driving force behind stress resistance is the ability to regulate emotions in a contextually appropriate and goal-directed manner (Gross, 2015; Waugh et al., 2011). While recent work suggests that there are likely to be a number of different strategies used during emotion regulation processes, and that these strategies may even be used simultaneously (Ford et al., 2019), two of the most commonly investigated in empirical settings are cognitive reappraisal and expressive suppression. Cognitive reappraisal (CR) refers to the process of deliberately changing an emotional response by deploying cognitive strategies that alter the meaning or relevance of a stimulus (Gross & John, 2003; Gross, 1998/2015). In contrast, expressive suppression refers to the process of inhibiting emotional responses elicited from a stimulus (Gross & John, 2003; Gross, 1998/2015). A large body of work now shows that cognitive reappraisal can effectively reduce subjective (Gross, 1998; Lieberman et al., 2011; Ray et al., 2010; Szasz et al., 2011; Wolgast et al., 2011), physiological (Delgado et al., 2008; Kim & Hamann, 2012; Ochsner & Gross, 2008; Shurick et al., 2012; Wolgast et al., 2011), and neural markers (Goldin et al., 2008; Ochsner et al., 2012; Picó-Pérez et al., 2017) of negative emotion experience in laboratory settings, while expressive suppression has typically been shown to be less effective at reducing negative emotional experience (Gross, 1998/2015; Gross & John, 2003; Goldin et al., 2008; Brans et al., 2013). Further, the tendency to engage in reappraisal in daily life—as measured by the Emotion Regulation Questionnaire (ERQ; Gross & John, 2003)—has also been shown to buffer individuals from the effects of daily life stress (Johnson et al., 2016). Specifically, those who reported more habitual use of reappraisal, but not suppression, tended to report less negative mood in response to daily stressors (Johnson et al., 2016). This suggests that while stressors can promote negative appraisals of one’s environment, the propensity to use reappraisal may be one factor that determines whether such biases emerge under stress.

Our hypothesis that reappraisal (but not suppression) may confer resilience against stress-induced negativity bias is informed by two decades of empirical work pointing to reappraisal as particularly effective in adaptively controlling emotional responses (Webb et al., 2012; see Gross, 2015 for review) and promoting positive psychological health (Aldao et al., 2010; Kring & Werner, 2004). We note, however, that despite this evidence, the utility of these two strategies are thought to be context-dependent—e.g., there may be situations in which reappraisal is not adaptive given the current circumstances, or suppression is preferred due to cognitive or environmental demands (or an entirely different regulation strategy is more appropriate). This suggests that, as more recently proposed (Bonanno & Burton, 2013; Ford & Troy, 2019; Gross, 2015; Troy, Shallcross, & Mauss, 2018), the most adaptive emotion regulation strategy value is ultimately the one that is contextually appropriate and allows an individual to achieve their desired emotional state. Nonetheless, given that the use of reappraisal has been found to be particularly well-suited to contexts marked by uncontrollable stress—that is, where individuals can control their responses to stressors rather than the stressor itself (Troy, Shallcross, & Mauss, 2018; Haines et al., 2016)—here, we hypothesize that more frequent use of reappraisal in daily life may render individuals less susceptible to the effects of stress on appraisals of ambiguity.

To examine this question, the present studies examined whether habitual reappraisal and suppression (as measured by ERQ) might serve as putative moderators of stress-related negativity bias. We note that our measure of valence bias is intended to index perceptions of emotionally ambiguous stimuli rather than the subjective emotional experience generated from these stimuli. We predicted that individuals who report more habitual reappraisal, but not suppression, will demonstrate a smaller shift toward negative perceptions of ambiguity after stress exposure. Since individuals that more frequently use reappraisal to regulate negativity may either be more motivated to experience positive emotions or have more experience successfully regulating negativity in daily life, we reasoned that these individuals may be more likely to circumvent the initial negative appraisal of ambiguity widely documented in the literature (Neta & Tong, 2016; Petro et al., 2018). As well as any potential increase in negativity bias that could arise through stress exposure (Brown et al., 2017). We first explored this question in the context of an existing data set from a study using a laboratory stress manipulation (Study 1) and further examined it in the context of real-world perceived stress imposed by the onset of the COVID-19 pandemic in a second, independent study (Study 2).

## Study 1

### Method

#### Participants

The initial sample included 52 participants recruited from the University of Nebraska-Lincoln and the surrounding community. An a priori power analysis conducted for our originally published study (Brown et al., 2017) used a between-group comparison of cortisol concentrations after a stressor described in previous work (*d* = -0.89; Raio et al., 2013). This power analysis identified a minimum of 21 participants per group which were necessary to replicate this cortisol comparison with 80% power, *α* = 0.05. Given that this was an existing data set, all analyses were constrained to the original sample size reported in our original paper (Brown et al., 2017). We note that this a priori power analysis was intended for detection of group-level cortisol responses, which was directly relevant to the central hypothesis in Brown et al. (2017), but as such may have left us somewhat underpowered for exploring individual differences in cognitive reappraisal. To address this, we provide complementary mixed effects analyses in supplementary material that leverage our repeated measures design to increase statistical power; this supplementary analysis yielded the same pattern of results (see Supplemental Materials, Tables S1 and S2).

Consistent with our previously published methods (Brown et al., 2017), six participants were excluded from the sample for the following reasons: non-normative ratings of clearly valenced faces (*n* = 1) using the same threshold of minimum 60% accuracy as in prior work (Neta & Tong, 2016), demonstrating cortisol changes that were more than two standard deviations from the group mean (*n* = 3), providing insufficient saliva for cortisol analysis (*n* = 1), and failing to complete both sessions (*n* = 1). Due to computer error, ERQ data was not recorded for three additional participants. Forty-three participants were included in this report: 20 participants (10 female; age range = 18–27; M, SD age = 20.35, 2.25; race = 20 White) that were randomly assigned to the stress group and 23 (11 female, age range = 18–35, M, SD age = 20.04, 3.57, race = 23 White) that were randomly assigned to the control group. Participants provided written informed consent at the start of each session. All procedures were approved by the University of Nebraska-Lincoln Committee for the Protection of Human Subjects (IRB approval #: 20151215793EP) and were in accordance with the 1964 Declaration of Helsinki.

#### Stimuli

As in prior work (Neta et al., 2009), stimuli included 48 pictures of faces with either an ambiguous valence (surprise, 24 pictures) or a clear (unambiguous) valence (angry and happy, 12 of each). All expressions were validated by a separate set of participants who labeled each expression; only faces correctly labeled more than 60% of the time were included. Notably, although some of the surprised expressions might be inherently more positive than others, the critical feature of this task is that all subjects rate the same set of faces and that there has been shown to be wide variability in ratings across subjects. Fourteen distinct identities were selected from the NimStim standardized facial expression stimulus set (Tottenham, Tanaka, Leon et al., 2009), and 20 identities were selected from the averaged Karolinska Directed Emotional Faces database (Lundqvist et al., 1998). Genders were represented equally, though each identity was not represented in all three expressions.

#### Procedure

Participants completed two sessions a week apart, as part of a study to examine the effects of acute stress exposure on valence bias (Brown et al., 2017). On day 1, participants completed the Emotion Regulation Questionnaire (ERQ), which is a 10-item questionnaire that measures the tendency to regulate one’s emotions using Cognitive Reappraisal or Expressive Suppression strategies (Gross & John, 2003). The ERQ measures responses on a 7-point scale ranging from strongly disagree to strongly agree, where higher numbers indicate increased use of a particular strategy. The ERQ has been shown to have acceptable to excellent levels of internal consistency reliability for both cognitive reappraisal (Cronbach's alpha = 0.82–0.90) and suppression (alpha = 0.76–0.80; Wiltink et al., 2011; Preece et al., 2021) and showed similar reliability estimates in our sample as well (ERQ Reappraisal: alpha = 0.88; ERQ Suppression: alpha = 0.75).

Participants then provided a saliva sample to assess day 1 baseline cortisol levels and completed the baseline valence bias task. On day 2, participants provided a saliva sample to assess day 2 baseline cortisol, followed by a stress manipulation consistent with procedures used by Raio et al. (2013). Specifically, participants in the stress group completed the cold-pressor task (Velasco et al., 1997), which involved submerging the forearm in ice water (0–4 °C, stress group) for three consecutive minutes. Participants provided a saliva sample 10 min after removing their arm from the water—when cortisol levels were beginning to peak—and then immediately proceeded to the valence bias task, so it would be completed during these cortisol elevations. Participants provided another saliva sample 50 min after removing their arm from the water, when cortisol levels were expected to return to baseline (to measure the likely recovery response; see Brown et al., 2017), and then completed the valence bias task one final time. Participants in the control group completed the task using warm water (~ 37 °C); all other saliva sampling procedures were identical to that of the stress group.

##### Valence Bias Task

The face stimuli were divided into three subsets of 16 faces. The 16 faces within each subset were presented four times in randomized order, for a total of 64 trials. Participants saw a different subset each of the 3 times they completed the valence bias task, and the order in which each subset was presented (at baseline, 10 min post-stressor, or 50 min post-stressor) was counterbalanced across all participants. MouseTracker software (Freeman & Ambady, 2010) was used to present the stimuli for 500 ms each and to record the mouse trajectories of each response as participants rated each face as positive or negative. We used mouse tracking because it is a valid index of response competition (Freeman et al., 2011) and because data from mouse tracking can be used to target different parts of the decision process (Hehman et al, 2015). Participants were instructed to rate the faces as quickly and accurately as possible, and the task did not advance until they made a response. Each trial was followed by as ISI varying from 500 to 8,000 ms. Trajectory data were recorded to test hypotheses specifically related to the effects of stress on valence bias and are reported in Brown et al. (2017). Exploratory analyses showed no relationship between mouse trajectories and ERQ scores.

### Results

#### Stress Manipulation Check (Cortisol Analysis)

Analysis of cortisol concentrations evidenced an effective stress induction as reported in more detail in our original report (Brown et al., 2017). Briefly, cortisol levels did not differ between the stress and control group on day 1, when baseline valence bias was measured (*t*, 41 = -1.15, *p* = 0.26, *d* = 0.35). On day 2, a group (stress, control) × time (baseline, 10 min post-stressor, 50 min post-stressor) repeated-measures ANOVA revealed a significant group × time interaction (*F*, 2, 82 = 4.61, *p* = 0.013, partial *η*^*2*^ = 0.10). Bonferroni-corrected post hoc comparisons showed that cortisol at 10 min post-stressor was significantly higher for the stress group (M, SD = 0.32, 0.19) than the control group (M, SD = 0.20, 0.13, 95% CI [0.02, 0.22], *p* = 0.02,), but not at day 2 baseline (95% CI [-0.05, 0.13], *p* = 0.40,) or 50 min post-stressor (95% CI [-0.03, 0.12], *p* = 0.20; see Table 1).

**Table 1:** Physiological and behavioral descriptives

		Baseline (day 1)		Baseline (day 2)		10 Minutes post-manipulation		50 Minutes post-manipulation	
		M	SD	M	SD	M	SD	M	SD
Cortisol concentrations (µg/dL) by group	Stress	0.26	0.16	0.26	0.15	0.32	0.19	0.25	0.11
	Control	0.22	0.10	0.22	0.14	0.20	0.13	0.21	0.12
Face ratings (% negativity) by expression condition	Stress								
	Angry	98.75	4.35	NA		99.69	1.40	99.69	1.40
	Happy	0.31	1.40	NA		0.94	3.06	2.81	7.44
	Surprise	70.11	19.00	NA		70.31	27.14	66.44	28.21
	Control								
	Angry	99.73	1.30	NA		98.37	6.59	98.37	4.70
	Happy	0.82	2.15	NA		0.54	1.80	3.80	15.62
	Surprise	67.64	21.58	NA		68.34	22.81	69.43	20.75

#### Valence Bias

The dependent measure used for the valence ratings of faces was percent negative ratings, or the percent of trials that a face was rated as negative out of the total number of ratings made for that expression condition. Valence bias scores for each participant were calculated as the percent negative ratings for the surprised faces only. Consistent with a large body of work examining valence bias in response to emotional ambiguity (Neta et al., 2009, 2013; Petro et al., 2018), ratings of happy and angry faces were not analyzed and served solely as a control to ensure that individuals were able to correctly identify clearly valenced facial expressions. Any deficits in this capacity indicated that surprise ratings may not reflect valence bias, but a broader deficit in facial expression recognition. As expected, participants consistently rated angry faces as negative and happy faces as positive, whereas ratings of surprise varied across individuals (see Table 1), consistent with previous work (Neta et al., 2009). The results of a linear regression show that neither reappraisal, nor suppression, was directly associated with valence bias (ERQ-R, *β* = 0.24, S.E. = 0.15, *t* = 1.58, *p* = 0.12; ERQ-S, *β* = 2.57, S.E. = 0.15, *t* = 0.85, *p* = 0.40). Because we were primarily interested in understanding variability in ratings in response to the stress manipulation, we focused our analyses on valence bias change scores (10 min post-stressor minus baseline), as previous work demonstrated an association between physiological indices of stress and more negative ratings during this time point (Brown et al., 2017).

#### Emotion Regulation Strategies and Increased Negativity

**Fig. 1:**
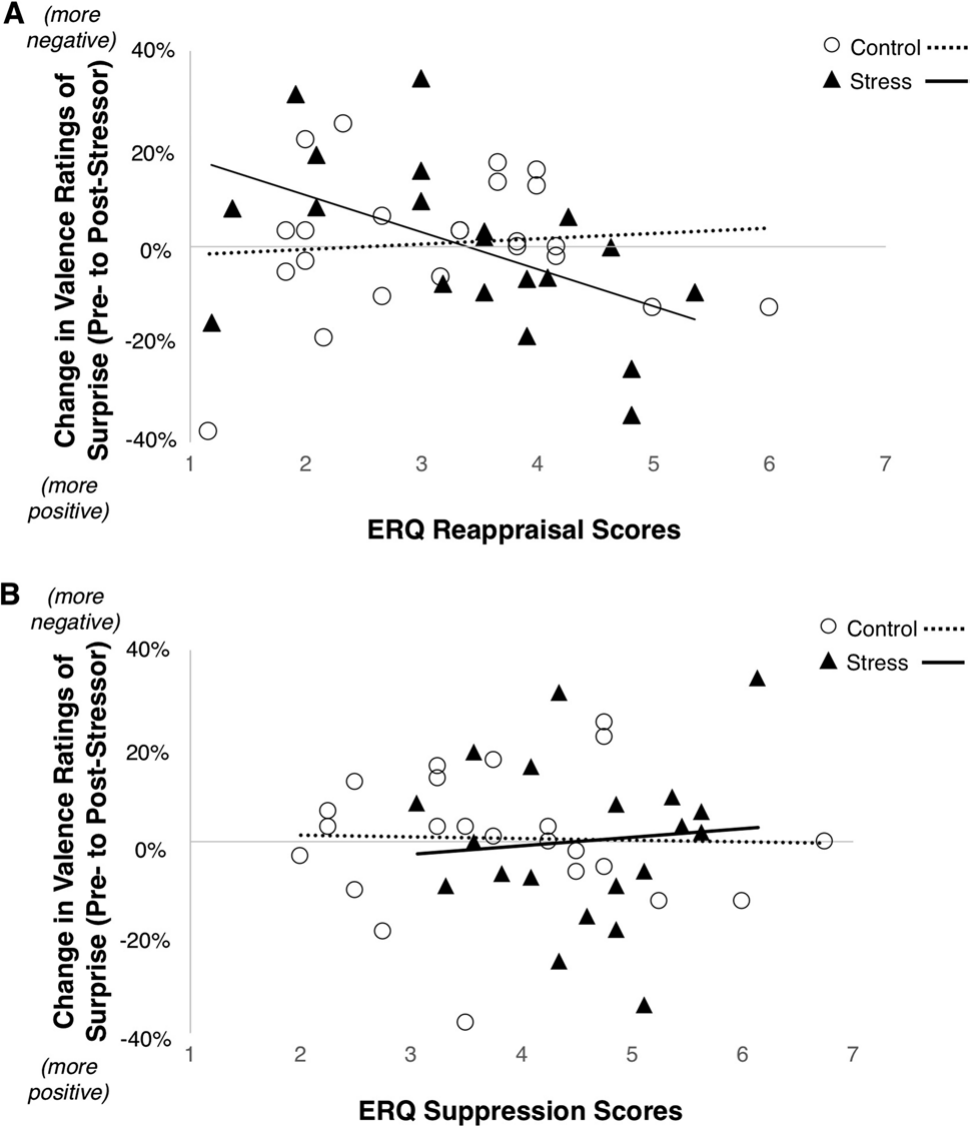
Relationship between regulation strategy and changes in valence ratings of surprised faces. **A** Change in valence ratings was negatively related with the ERQ Reappraisal score in the stress group (*β* = -.59, 95% CI [-1.05, -0.14], *t,* 39 = -2.63, *p* = .012) but not controls (*β* = .08, 95% CI [-0.31, 0.47], *t,* 39 = .41, *p* = .69), such that participants in the stress group who used reappraisal more habitually showed less of an increase in negativity. **B** The change in valence ratings showed no significant relationship with the ERQ Suppression score in either group (*p*s > .68)

We first conducted two separate regression models predicting the change in valence bias using ERQ Reappraisal and ERQ Suppression as predictors in each of the models. Change in valence bias and ERQ scores was scaled prior to analysis (i.e., mean-centered and divided by standard deviation), but the group variable was not, meaning partially standardized estimates are reported. Specifically, we conducted a regression on valence bias change scores with group (stress, control), ERQ Reappraisal, and their interaction as predictors. While we observed no main effect of the stress manipulation (group) on valence bias (i.e., no change in valence bias overall pre- to post-stressor; Table 1), there was a significant group × ERQ Reappraisal interaction (*β* = -0.67, 95% CI [-1.28, -0.07], *t,* 39 = -2.26, *p* = 0.03), such that greater ERQ Reappraisal scores were negatively associated with valence bias change scores only for the stress group (*β* = -0.59, 95% CI [-1.05, -0.14], *t,* 39 = -2.63, *p* = 0.012), and not for the control group (*β* = 0.08, 95% CI [-0.31, 0.47], *t, *39 = 0.41, *p* = 0.69; Fig. 1A). That is, participants in the stress group who reported using reappraisal more often in daily life showed less of an increase in negative perceptions of ambiguity.

In contrast to the ERQ Reappraisal model, the model that included group (stress, control), ERQ Suppression, and their interaction as predictors was not significant (*F,* 3, 39 = 0.07, *p* = 0.98, multiple *R*^*2*^ = 0.01), and there was no interaction effect in the model (*β* = 0.15, 95% CI [-0.60, 0.91], *t,* 39 = -0.42, *p* = 0.68). No relationships were observed between valence bias change and the ERQ Suppression score for the control group (*β* = -0.02, 95% CI [-0.42, 0.38], *t,* 39 = -0.13, *p* = 0.90) or stress group (*β* = 0.13, 95% CI [-0.50, 0.77], *t,* 39 = 0.42, *p* = 0.68; Fig. 1B). Overall, the ERQ Reappraisal model explained 15.43% of the variance, and the ERQ Suppression model explained 0.51% of the variance.

Finally, in order to examine the unique contribution of ERQ Reappraisal on valence bias change (above and beyond that of ERQ Suppression), we conducted a third regression on valence bias change scores including all predictors in the same model: group (stress, control), ERQ Reappraisal, ERQ Suppression, and their two interactions (*F, *7, 35 = 2.21, *p* = 0.06, multiple *R*^*2*^ = 0.31). This analysis revealed a significant group × ERQ Reappraisal interaction (*β* = -0.85, 95% CI [-1.50, -0.19], *t,* 39 = -2.63, *p* = 0.01), but no ERQ Suppression x group interaction (*β* = 0.28, 95% CI [-0.42, 0.98] *t,* 39 = 0.80, *p* = 0.43). These results support our prediction of a selective relationship between reappraisal—but not suppression—and valence bias change and suggest that this effect was moderated by group such that the effect was only significant for those undergoing a stress induction.

Sensitivity analyses in G*Power revealed that we could reasonably detect a minimum detectable effect size of f^2 ^ = 0.28 for a multiple regression with three predictors (alpha = 0.05, power = 0.8). Although we acknowledge that these effect sizes are larger than the reported interaction effect here (f_2_ = 0.13), it has also been shown that effects estimated from a single study are noisy estimates of the true population effect (see Gelman, 2018). Having said that, we sought to replicate these results using linear mixed effects models, which enabled us to leverage our repeated measures design to increase power and provide additional support for our findings (Table S1 and S2). We fit a linear mixed effects model with a random intercept for each subject, to account for within-subject variance, and fixed effects of ERQ (suppression or reappraisal), time (pre- to post-stressor), group (stress, control), and their interactions. A significant three-way interaction, consistent with our original findings, was observed in this more highly powered model for ERQ Reappraisal (*β* = 0.46, [0.06 – 0.86], *p* = 0.02) but not ERQ Suppression (*β* = 0.11, [-0.39 – 0.61], *p* = 0.67).

#### Emotion Regulation Strategies and Increased Cortisol

We conducted similar regression models predicting change in cortisol levels (rather than change in valence bias) from day 2 baseline to post-stressor, using ERQ Reappraisal (*F,* 3, 39 = 5.33, *p* = 0.004, multiple *R*^2^ = 0.29) and ERQ Suppression (*F,* 3, 39 = 4.34, *p* = 0.01, multiple *R*^2^ = 0.25), respectively. No interaction effects emerged in the model using group (stress, control), ERQ Reappraisal, and their interaction as predictors (*β* = -0.42, 95% CI [-0.98, 0.12], *t,* 39 = -1.57, *p* = 0.13), nor in the model with group (stress, control), ERQ Suppression, and their interaction as predictors (*β* = -0.25, 95% CI [-0.91, 0.39], *t*, 39 = -0.80, *p* = 0.43).

## Study 2

Study 1 revealed that increased negativity bias after exposure to stress is conditional upon self-reported use of reappraisal strategies to regulate one’s emotions. That is, those who engage in less reappraisal showed a stress-related negativity bias, whereas those who engage in more reappraisal did not show such a bias. However, our initial assessment of this relationship occurred within a controlled laboratory setting and in a relatively small sample. Thus, to probe whether such an effect persists in real-world stressful contexts, and to directly test if reappraisal use moderates the effects of stress on negativity bias, we next sought to replicate and extend our findings in an independent sample experiencing stress during the onset of the COVID-19 pandemic.

### Method

#### Participants

Participants were recruited for an online study that measured valence bias before and after the start of the COVID-19 pandemic, using Amazon’s Mechanical Turk (M-Turk; Horton et al., 2011). Participants completed two sessions in Gorilla Experiment Builder (Anwyl-Irvine et al., 2020), one between October 2019 and January 2020 (before the onset of the COVID-19 pandemic) and a second session between April and May 2020 (after the pandemic onset), as part of a study to understand the effects of real-world perceived stress on valence bias. Data were collected over the span of several months due to strict inclusion criteria. Only participants aged 18 or older, that spoke English as their native language, and did not have a history of psychological or neurological disorders were allowed to continue beyond a demographic screener (*n* = 679 excluded). Additionally, participants were rejected from the task in the event that they did not complete it within 1 h and 30 min (*n* = 172 excluded). This final criterion was applied because there is evidence to suggest that unreasonably long times spent on surveys may indicate poor data quality (e.g., using virtual private networks to bypass geolocation requirements; Kennedy et al., 2020) and pilot testing revealed that the task could be consistently completed in approximately 30 min. Of the 229 participants included in the initial wave of data collection before the onset of the pandemic (Harp et al., 2020; 122 female; age range = 18–76 years; mean (SD) age = 44.77 (14.43); race distribution: 15 Asian, 20 Black, 177 White, 5 other, and 12 unknown), 105 participants volunteered to participate in a follow-up after the onset of the pandemic. According to an a priori power analysis, a minimum of 77 participants were necessary for a moderation analysis with 80% power, *α* = 0.05 and an *f*^*2*^ = 0.15.

As in Study 1 and consistent with previous work (Neta & Tong, 2016; Neta & Whalen, 2010; Neta et al., 2013), six participants were excluded from the follow-up sample for non-normative ratings of clearly valenced faces (i.e., accuracy for valence ratings of angry and/or happy faces was below 60%) to ensure an accurate representation of the bias in response to ambiguity. Further, two participants were removed for scoring more than 3 SDs below the mean on the ERQ (Reappraisal), since this was the primary construct of interest and would serve as our moderator variable. Our final sample consisted of 97 participants (53 female; age range at the first session = 21–76; M (SD) age = 47.58 (13.73); race distribution: 5 Asian, 8 Black, 76 White, 1 other, and 7 unknown). Participants provided informed consent at the start of each session. All procedures were approved by the University of Nebraska-Lincoln Committee for the Protection of Human Subjects (IRB approval #: 20200520425EP) and were in accordance with the 1964 Declaration of Helsinki.

#### Stimuli

Three task blocks (faces, scenes, and words) were used to assess valence bias. As in previous work (Neta et al., 2013), the face and scene task blocks included 24 ambiguous images and 24 clear images (12 positive and 12 negative). The facial expressions were selected from the NimStim (Tottenham et al., 2011) and Karolinska Directed Emotional Faces (Lundqvist et al., 1998) sets, and the scenes were selected from the International Affective Picture System (IAPS; Lang et al., 2008). For the words block, the 32 ambiguous, 16 positive, and 16 negative words were used (Harp et al., 2020). However, for the purposes of this experiment in replicating and extending findings from Study 1 that used only face stimuli, here we focus only on responses to the face blocks. As in Study 1, ratings of happy and angry faces were not of interest above and beyond serving as anchors to ensure a reliable measure of valence bias under emotional ambiguity (i.e., surprise).

#### Procedure

Participants completed two sessions, one between October 2019 and January 2020 (before the onset of the COVID-19 pandemic) and a second session between April and May 2020 (after the pandemic onset), as part of a study to understand the effects of real-world stress on valence bias. During Session 1, participants completed the valence bias task on face, scene, and word stimuli, where each stimulus category was presented in separate blocks (2 total blocks for each category). The order of blocks was counterbalanced across participants, but one block of each category was always presented before presenting the second block for each category (Harp et al., 2020). Each block included 50% stimuli that were ambiguous, and 50% that were clearly valenced (25% clearly positive and 25% clearly negative), as in previous work (Neta et al., 2013). Note that, for the purposes of this report in extending the findings from Brown et al. (2017), we focus on the valence rating responses to ambiguous face stimuli (clear stimuli were presented primarily to ensure that participants were performing the task accurately). During Session 2, participants completed the same valence bias task again in a new counterbalanced order and then completed a series of self-report surveys including the Emotion Regulation Questionnaire (ERQ; Gross & John, 2003). As in Study 1, the ERQ showed adequate reliability in our sample for both ERQ Reappraisal (alpha = 0.86) and ERQ Suppression (alpha = 0.83).

Participants also completed the Perceived Stress Scale (PSS; Cohen et al., 1983), a widely used and well-validated measure of self-reported perceived stress experienced over the previous month. The PSS is a 10-item questionnaire for which perceptions of stress—or more specifically subjective distress experienced from stressors—is measured by probing how uncontrollable, unpredictable, and overloaded participants have felt over the last month, with responses provided on a 4-point scale ranging from never to very often. The PSS has been found to have acceptable to excellent levels of internal consistency reliability (*α* = 0.78; Cohen & Williamson, 1988; *α* = 0.87; Baik et al., 2019) and showed good reliability in our sample (alpha = 0.92). Self-report surveys were administered after the face ratings since valence bias is known to be highly sensitive to transient changes in affective state (Brown et al., 2017; Neta et al., 2011; Neta, Cantelon, Mahoney, et al., 2017; Neta, Cantelon, Haga, et al., 2017), and we wanted to avoid any priming effects that could potentially arise from the affective scales participants were completing.

### Results

Given that stress exposure promoted negative appraisals of ambiguous stimuli among those with lower self-reported reappraisal tendencies in Study 1, here we examined the extent to which this effect generalized to real-world stressful contexts (i.e., perceived stress during the COVID pandemic). We capitalized on the continuous nature of participants’ perceived stress scores—unlike Study 1, where stress group assignment was binary—by testing whether reappraisal tendency moderated the effect of perceived stress on change in negativity bias. Our moderation model was conducted using R (R Core Team, 2019), where change in negativity bias (a difference score in ratings of surprised faces after > before the onset of the COVID pandemic) was included as the outcome variable, with Perceived Stress Scale (PSS) scores as the predictor and ERQ Reappraisal as a moderator of the relationship between these variables. ERQ Reappraisal, perceived stress, and change in negativity bias were scaled (i.e., mean-centered and divided by the standard deviation) prior to analysis and all reported effect estimates are standardized. Table 2 reports the descriptive statistics for each variable.

**Table 2:** Descriptive statistics of observed variables

	M (SD)	Sample range	Possible range
Change in negativity bias (valence bias ratings after > before the onset of the COVID pandemic)*	11.75% (29.62)	-52.17 to + 100%	-100 to + 100%
Reappraisal score (ERQ-R)	5.18 (1.03)	1.83 to 7	1 to 7
Suppression score (ERQ-S)	3.52 (1.44)	1 to 7	1 to 7
Perceived stress score (PSS)	15.16 (7.96)	1 to 32	0 to 40

^*^Note: Negative values in change in negativity bias denote a shift toward more positive ratings of surprised faces after compared to before the onsets of the COVID-19 pandemic; positive values denote a shift toward more negative ratings

The overall model was marginally significant in accounting for 7.8% of the variance (*F,* 3, 93 = 2.61, *p* = 0.056, multiple *R*^2^ = 0.08). The interaction uniquely accounted for 5.2% of the variance (*β* = -0.24, 95% CI [-0.46, -0.03], *t,* 93 = -2.29, *p* = 0.02), indicating that the relationship between change in negativity bias and perceived stress was indeed moderated by reappraisal tendency (ERQ Reappraisal). The conditional effect of perceived stress on change in negativity bias was significant at one standard deviation below the mean of cognitive reappraisal (*β* = 0.40, 95% CI [0.11, 0.69], *t,* 93 = 2.75, *p* = 0.007), but not at the mean (*β* = 0.15, 95% CI [-0.05, 0.36], *t,* 93 = 1.49, *p* = 0.14), or at one standard deviation above the mean (*β* =  − 0.09, 95% CI [-0.40, 0.21], *t,* 93 =  − 0.61, *p* = 0.54).

**Fig. 2:**
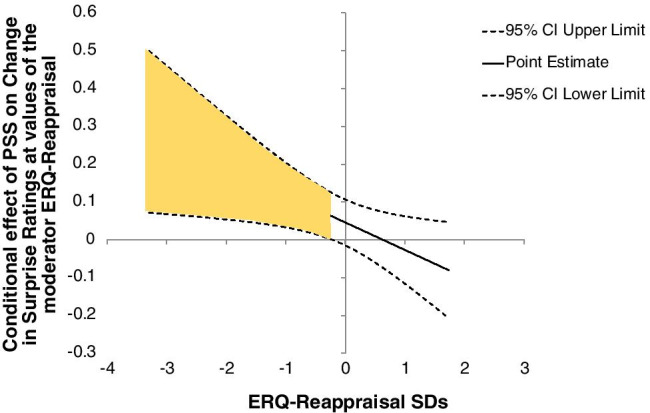
Relationship between valence bias and perceived stress as a function of ERQ-R score. The conditional effect of perceived stress during the COVID pandemic on valence bias change is plotted for a range of ERQ-R scores. In individuals with low reappraisal tendency, perceived stress increased negative ratings of ambiguous facial expressions, while in individuals with high reappraisal tendency, this stress-related increase in negativity bias was not observed

To further probe the significant interaction, we conducted a regions of significance analysis such that conditional effects of perceived stress on change in negativity bias were estimated at all observed levels of ERQ Reappraisal (i.e., scores ranging from 1.83 to 7), and the significance of those conditional effects were examined. Perceived stress was associated with a greater increase in negativity bias for individuals with an ERQ Reappraisal scores -0.22 below the mean and lower (i.e., scores ranging from 1.83 to 4.96; see Fig. 2). That is, participants experiencing higher perceived stress after the onset of the COVID pandemic who also report lower reappraisal tendency showed more of an increase in negative perceptions of ambiguity. Mirroring the findings of Study 1, there was no main effect of perceived stress on valence bias as assessed using linear regression (*β* = 4.56, S.E. = 3.05, *t* = 1.49, *p* = 0.14).

We next assessed whether a similar effect was observed using suppression tendency (ERQ-S) rather than reappraisal. A comparable moderation analysis was not significant (*F,* 3, 93 = 0.91, *p* = 0.44, multiple *R*^2^ = 0.03) and revealed no significant interaction between ERQ suppression and perceived stress (*β* = 0.00, 95% CI [-0.20, 0.21], *t* = 0.02, *p* = 0.99). In other words, ERQ suppression does not appear to moderate the relationship between perceived stress and valence bias change. Further, the results of a linear regression revealed that suppression was not directly associated with valence bias in Study 2 (*β* = 2.57, S.E. = 3.12, *t* = 0.83, *p* = 0.41), nor did suppression interact with perceived stress scores to predict change in valence bias (*β* = 0.05, S.E. = 3.07, *t* = 0.02, *p* = 0.99).

Finally, sensitivity analyses in G*Power revealed that we could reasonably detect a minimum detectable effect size of *f*^*2*^ = 0.12 for a multiple regression with three predictors (alpha = 0.05, power = 0.8). Given that these effect sizes are larger than the reported interaction effect here (*f*^*2*^ = 0.06), we again sought to replicate our findings using a linear mixed effects models, which enabled us to leverage our repeated measures design to increase power and provide additional support for our findings (see Supplemental Materials, Table S3). Specifically, we tested our hypothesis that reappraisal (ERQ Reappraisal) moderates the relationship between perceived stress (PSS) and shifts in percent negative ratings from before to after the beginning of the pandemic (time: pre- vs. post-pandemic). This model allows us the opportunity to make use of data from both time points, rather than reducing change over time to a single data point for each subject. Specifically, we tested a model with random intercepts for each subject, to account for subject-level variance, and then tested for fixed effects of ERQ Reappraisal, PSS, and time as well as their interactions. A significant three-way interaction revealed that our findings are robust to this more highly powered model (*β* = -0.27, [-0.51, -0.04], *p* = 0.02).

## Discussion

Our findings demonstrate an important role for reappraisal tendencies as a moderator of negativity bias after stress exposure. Across two independent studies, we found that increases in negative perceptions of ambiguous social stimuli after stress exposure were conditional on the habitual use of reappraisal. Specifically, participants who reported using less reappraisal showed an increased negativity bias after stress exposure, whereas those who engage in more reappraisal did not show such a bias. We also replicated these findings outside a laboratory setting in the context of a real-world perceived stress during the COVID-19 pandemic. These findings suggest that those who more often engage in reappraisal may experience a milder impact of stress (Jamieson et al., 2012) on valence bias, perhaps prompting a less negative emotional experience following stress (Fredrickson et al., 2003). Importantly, we observed no main effect of stress on valence ratings across both studies, suggesting that it was not stress exposure per se, but its interaction with reappraisal tendency that determined change in negativity bias. These findings are also consistent with recent work showing that cognitive forms of emotion regulation mediate the association between ventromedial prefrontal cortex activity during a stressor and more positive emotions during stress recovery (Yang et al., 2018).

In Study 1, although habitual reappraisal mitigated the negative behavioral consequences of stress, it showed no impact on physiological stress reactivity (changes in cortisol following a stressor). Interestingly, the literature shows inconsistent findings relating reappraisal and cortisol reactivity. For example, some studies demonstrate that reappraisal during a laboratory-induced stressor is associated with greater cortisol reactivity, while others showed no effect or have found that habitual reappraisal was associated with less cortisol reactivity (see Krkovic et al., 2018 for a review). Notably, the latter finding measures responses to a stressor that was qualitatively different from laboratory-based inductions in that it was voluntary (i.e., skydiving). Indeed, participants who volunteer for such an unpredictable and stressful experience may already appraise ambiguity in a more positive light to begin with (Crum et al., 2020). These strategies, when activated during an acute stressor, could help them arrive at positive perceptions of ambiguity.

In contrast to Study 1, in which stress exposure was experimentally induced, Study 2 assessed whether this effect of reappraisal tendency generalized to real-world perceived stress. By measuring valence bias change in participants before and after the start of the COVID pandemic, we were able to leverage the effects of a widespread societal stressor on participants that varied depending on each individuals’ subjectively perceived stress. This afforded the opportunity to test whether these results replicated using a continuous measure of perceived stress, rather than within a laboratory setting. In doing so, we observed that habitual reappraisal moderated the effect of perceived stress on the change in bias, such that lower levels of reappraisal use revealed an increase in perceptions of negativity under higher perceived stress. On the other hand, this effect was not apparent at higher levels of reappraisal, suggesting that deficits in reappraisal tendencies may place individuals at a heightened risk for negativity bias in the presence of stress. In contrast, habitual use of expressive suppression to regulate emotion did not reveal the same pattern of results. Collectively, these findings suggest that habitual use of cognitive strategies to change one’s emotional state (reappraisal) may generalize to attenuating stress-induced negativity bias of ambiguous emotional stimuli.

It should be noted that while the use of reappraisal in laboratory settings has been shown to be associated with reduced negative emotional experience (Gross, 1998; Lieberman et al., 2011; Ray et al., 2010; Szasz et al., 2011; Wolgast et al., 2011), a growing body of work has shown the use of reappraisal in daily life is reliably related to increased positive affect (Blaxton & Bergeman, 2017; Brans et al., 2013; Brockman et al., 2017; Kuppens et al., 2010; Nezlek & Kuppens, 2008; Pavani et al., 2016; Richardson, 2017; Troy, Shallcross, & Mauss, 2018; Troy, Shallcross, Brunner, et al., 2018). Since our face rating task quantifies valence bias on a continuum from negative to positive, any attenuation in negativity bias is inherently an increase in positivity bias. Thus, our results are consistent with this body of work and can be interpreted as showing that higher levels of habitual reappraisal reduce negativity bias—or inversely, increase positivity bias—when evaluating emotional ambiguity.

There are a number of potential mechanisms through which habitual reappraisal may shape individual differences regarding stress-related negativity bias. First, we note that it is unlikely that individuals are deliberately recruiting reappraisal strategies to regulate valence bias under stress. Instead, we propose that this effect points to a mechanism whereby individuals in the practice of regulating potential negativity through reappraisal may more spontaneously override the initial negative appraisals that arise when confronting ambiguity and, more specifically, override negativity biases that can potentially be imposed by exposure to stress. This mechanism may emerge through learning, such that those who use reappraisal and find it successfully alleviates negative emotional states are reinforced to continue using this strategy when encountering stressors that can potentially increase negativity bias. This account is consistent with recent extensions of the process model of emotion regulation (see Gross, 2015), which proposes that the use of reappraisal arises through a valuation process that is informed by an evaluation of how useful reappraisal will be given the contextual and environmental demands on an individual (Gross, 2015), and presumably what strategies have worked in similar situations in the past (Etkin et al., 2016). While our self-report measure does not allow us to index the stage at which our participants determine reappraisal might have been a suitable strategy or how they navigated this dynamic process (Gross, 2015), this learning and valuation mechanism is consistent with the notion that those who find cognitive emotion regulation strategies valuable may be more inclined to use reappraisal in a goal-directed manner in the future.

Alternatively, this mechanism could arise through a motivational account—that is, more frequent use of reappraisal reflects an individual’s desire to appraise affective stimuli in a positive manner in order to foster positive emotional experiences. This motivational tendency may subsequently generalize to reduced negativity when appraising ambiguous stimuli, even under stressful or challenging circumstances. Such an account is consistent with the fact that while tendencies to engage in more active emotion regulation strategies confer psychological resilience (Aldao et al., 2010; Gross, 2015; Kring & Werner, 2004; Webb et al., 2012), these constructs may proceed in a recursive manner, such that more psychologically resilient individuals may also be more inclined to use cognitive strategies to control emotional responses. Finally, these learning and motivational mechanisms need not be mutually exclusive—resilient individuals that tend to use reappraisal because they are motivated to achieve a particular emotional experience may then find these strategies more efficacious, reinforcing them for increased use in the future. Characterizing how motivational, learning, and valuation mechanisms interact and shape the use of cognitive emotion regulation strategies in daily life, as well as how they drive individual differences in perceptions of ambiguity, will be important for advancing future theoretical and empirical work on this topic.

A number of limitations should be noted for future work. Most notably, the sample sizes of both Study 1 and Study 2 were quite constrained. Study 1 was constrained to the existing sample size of our originally published study (Brown et al., 2017), and Study 2 was constrained by the number of participants that agreed to return and participate in the second session after the onset of the pandemic. Given the unforeseeable nature of the pandemic and the inability to notify participants of the subsequent sessions during the initial data collection, there was substantial attrition from the original study (Harp et al., 2020). Despite these sample size limitations, however, our mixed-method design across two studies does provide conceptual replication of the effect both within and outside the laboratory, suggestive of a robust effect that generalizes to multiple contexts. Additionally, we sought to address this concern in these studies by leveraging our repeated measures design to increase power in supplemental analyses for each study (Supplemental Materials, Tables S1, S2, and S3) and found that our findings were robust to this more highly powered model for each study. Nonetheless, future work should seek to replicate these effects in larger samples.

Future work may also seek to replicate these effects using alternative stress manipulation techniques (i.e., social-evaluative stressors such as the Trier Social Stress Test) to provide further evidence of the generalizability of the effect. Here, we chose to use a physiological stressor in our previous study on stress and valence bias (Brown et al., 2017) and, in this current work, to test how reappraisal potentially interacts with these stress effects. Indeed, we chose a physiological stressor because we sought to test whether the physiological effects of stress were robust enough to generalize to a social evaluation task even when the stressor itself was not socially evaluative. However, given the pervasive nature of social-evaluative stressors in daily life, it would be both interesting and important for future work to test how the negative social evaluation inherent in many social stressors affects evaluations of ambiguous social stimuli and whether the protective effect of reappraisal translates to the social domain.

Study 2 was conducted using a longitudinal design, rendering it difficult to retain our entire original sample size. Study 2 was also conducted online, which afforded us the opportunity to reach a more representative sample of individuals affected by the COVID pandemic. Additionally, we note that our final sample was heavily skewed toward white participants; thus, further research should investigate whether these results generalize to more diverse samples. More specifically, it will be important for future work to explore whether an increasingly diverse sample show similar effects as those seen here, especially given that it may be adaptive for minority group members to maintain a vigilant approach toward ambiguous facial expressions of majority group members, as has been shown in the context of interaction partners during interracial encounters (West et al., 2017). Finally, we note that in Study 2, we used the PSS to index perceived stress during the COVID pandemic; however, the PSS is ultimately a measure of subjective distress experienced from recent stressors rather than a definitive account of stressor exposure per se. Future work may seek to use more expansive stressor inventories or ecological momentary assessment (EMA) to probe how real-world stressors and reappraisal tendency affect valence bias.

It should be noted that while habitual use of reappraisal was protective against stress-induced negativity bias, cognitive reappraisal actually represents a number of different cognitive strategies that alter the meaning or relevance of an emotionally charged stimulus or situation (Gross, 2015). Cognitive reappraisal can be achieved by deliberately changing the way a stimulus is interpreted, by diminishing the self-relevance of a stimulus, by distancing oneself from an emotionally-charged situation (Gross, 1998/2015), or by adopting a mindset that reframes a stressor in a more positive, adaptive manner (Crum et al., 2020; Jamieson et al., 2018). Since the self-report instrument used here (ERQ) does not dissociate between these different sub-strategies of reappraisal, we were unable to identify exactly which feature of cognitive reappraisal participants used or what particular factors fed into this decision. Thus, a final important avenue for future research will be to identify the specific reappraisal strategy individuals are using in daily life, as well as what contextual, social, or emotional factors (external or internal) informed this decision. Such work will be critical to better understand the mechanisms that give rise to these protective effects of reappraisal on individual differences in valence bias, particularly when faced with a variety of stressors.

## Supplementary Information

Below is the link to the electronic supplementary material.

Supplementary file1 (PDF 39 kb)

## Additional Information

### Data Availability

Data from Study 1 cannot be shared publicly because the participants consented to the report of the data in terms of group means, and not individualized data. The University of Nebraska-Lincoln Institutional Review Board completed review of the original research protocol including data sharing restrictions. Data requests can be sent to David Clausen (dclausen2@unl.edu, 402–472-3366), the current contracts manager in the Office of Sponsored Programs at University of Nebraska-Lincoln. All data sharing requests are first subject to a contract agreement through the Office of Sponsored Programs. Data from Study 2 has been deposited into a publically available repository: https://osf.io/3e8x7/.
