# Supplementary material for: Reappraisal—but not Suppression—Tendencies Determine Negativity Bias After Laboratory and Real-World Stress Exposure
Source: Affect Sci. 2021 Oct 22;2(4):455–67. doi: 10.1007/s42761-021-00059-5 (PMC8531907; doi:10.1007/s42761-021-00059-5)
Supplement: Supplementary file 1 — Supplementary file1 (PDF 39 kb) [file 42761_2021_59_MOESM1_ESM.pdf]

Supplemental Table S1. Linear mixed effects analysis of Study 1 (ERQ-CR).

	Percent Negative Ratings (Surprise Faces)		
Predictors	*Partially Standardized Estimates*	*CI*	*p*
(Intercept)	0.05	-0.37 – 0.46	.818
ERQ CR	0.22	-0.22 – 0.65	.330
Time	0.01	-0.28 – 0.30	.952
Group	-0.11	-0.68 – 0.46	.702
ERQ CR x Time	-0.41	-0.71 – 0.10	**.008**
ERQ CR x Group	0.28	-0.30 – 0.85	.344
Time x Group	0.02	-0.37 – 0.42	.911
ERQ CR x Time x Group	0.46	0.06 – 0.86	**.024**
**Random Effects**			
σ^2	0.22		
$\tau_{00\ Subject}$	0.68		
ICC	0.76		
$N_{Subject}$	43		
Observations	86		
Marginal R^2 / Conditional R^2	0.165 / 0.798		

Supplemental Table S2. Linear mixed effects analysis of Study 1 (ERQ-ES)

	Percent Negative Ratings (Surprise Faces)		
Predictors	*Partially Standardized Estimates*	*CI*	*p*
(Intercept)	0.01	-0.42 – 0.44	0.974
ERQ ES	0.25	-0.13 – 0.63	0.198
Time	0.03	-0.27 – 0.33	0.861
Group	-0.01	-0.65 – 0.64	0.986
ERQ ES * Time	-0.02	-0.28 – 0.25	0.900
ERQ ES * Group	-0.10	-0.82 – 0.61	0.780
Time * Group	-0.05	-0.50 – 0.41	0.841
ERQ ES * Time * Group	0.11	-0.39 – 0.61	0.674
**Random Effects**			
σ^2	0.26		
$\tau_{00\ Subject}$	0.78		
ICC	0.75		
$N_{Subject}$	43		
Observations	86		
Marginal R^2 / Conditional R^2	0.046 / 0.765		

Supplemental Table S3. Linear mixed effects analysis of Study 2.

	Percent Negative Ratings (Surprise Faces)		
Predictors	*Partially Standardized Estimates*	*95% CI*	*p*
(Intercept)	-0.19	-0.39 – 0.00	.055
ERQ CR	-0.05	-0.25 – 0.15	.622
Time	0.40	0.17 – 0.62	**< .001**
PSS	-0.08	-0.27 – 0.12	.454
ERQ CR x Time	0.08	-0.15 – 0.30	.508
ERQ CR x PSS	0.16	-0.04 – 0.37	.119
Time x PSS	0.17	-0.05 – 0.40	.137
ERQ CR x Time x PSS	-0.27	-0.51 - -0.04	**.022**
**Random Effects**			
σ^2	0.61		
$\tau_{00\ Subject}$	0.34		
ICC	0.36		
$N_{Subject}$	97		
Observations	194		
Marginal R^2 / Conditional R^2	0.074 / 0.407		
